# Modeling methicillin-resistant *Staphylococcus aureus* in hospitals: Transmission dynamics, antibiotic usage and its history

**DOI:** 10.1186/1742-4682-9-25

**Published:** 2012-06-27

**Authors:** Farida Chamchod, Shigui Ruan

**Affiliations:** 1 Department of Mathematics, University of Miami, Coral Gables, FL 33124-4250, USA

## Abstract

**Background:**

Methicillin-resistant *Staphylococcus aureus* (MRSA) is endemic in many hospital settings, posing substantial threats and economic burdens worldwide.

**Methods:**

We propose mathematical models to investigate the transmission dynamics of MRSA and determine factors that influence the prevalence of MRSA infection when antibiotics are given to patients to treat or prevent infections with either MRSA itself or other bacterial pathogens.

**Results:**

Our results suggest that: (*i*) MRSA always persists in the hospital when colonized and infected patients are admitted; (*ii*) the longer the duration of treatment of infected patients and the lower the probability of successful treatment will increase the prevalence of MRSA infection; (*iii*) the longer the duration of contamination of health care workers (HCWs) and the more their contacts with patients may increase the prevalence of MRSA infection; (*iv*) possible ways to control the prevalence of MRSA infection include treating patients with antibiotic history as quickly and efficiently as possible, screening and isolating colonized and infected patients at admission, and compliance with strict hand-washing rules by HCWs.

**Conclusion:**

Our modeling studies offer an approach to investigating MRSA infection in hospital settings and the impact of antibiotic history on the incidence of infection. Our findings suggest important influences on the prevalence of MRSA infection which may be useful in designing control policies.

## Background

Methicillin-resistant *Staphylococcus aureus* (MRSA) is a major nosocomial pathogen that poses substantial threats and economic burdens to patients and hospitals globally. MRSA infection increases the risk of mortality, the length of hospital stay of patients, and extra costs of treatment and a control program for patients and hospital settings [1].

Although MRSA can be transmitted via contaminated objects, that is not the most common means of transmission [2,3]. Rather, transmission from patient to patient occurs via the hands of health care workers (HCWs) in hospitals. Patients may be categorized into three disease statuses: uncolonized, colonized, and infected with MRSA [4]. MRSA causes sequelae such as sepsis, abscesses, wound infection, skin and soft tissue infection, and bloodstream infection. However, it can also colonize healthy humans without causing infection. Many parts of the body can be colonized such as the axillae, perineum, groin, rectum, skin, and anterior nares. In fact, a third of humans are asymptomatic nasal carriers of *Staphylococcus aureus*[5,6]. The risk of colonization increases after contact with colonized and infected individuals [7]. Furthermore, it is more likely that individuals persistently colonized with MRSA will develop infection than those with short-term colonization [8,9].

Antibiotics are widely prescribed to inhibit bacterial infections. They help to kill bacteria or prevent them from reproducing. However, because bacteria are capable of multiplying rapidly and transferring plasmids, the use of antibiotics may act as an environmental pressure on bacteria to select for resistance mutations, and the resistance trait can be transmitted to their progeny and other recipients by vertical and horizontal gene transfer [10]. Hence, the use of antibiotics may predispose individuals to acquire resistant elements. Consequently, treating infected individuals may become increasingly difficult, especially patients who have previously received antibiotic treatment. The ability to acquire resistance has been noted particularly in *S. aureus*. Antibiotic treatment of individuals infected with MRSA has been shown to be associated with skin infection, colonization, and treatment failure [8,11-14]. In fact, a clear association between antibiotic exposure and MRSA isolation has been observed [12].

Various control methods have been suggested and implemented to overcome the spread of MRSA in hospital settings, including hand-washing compliance, staff cohorting, isolation of infected patients, use of rapid screening tests to identify acquisition of MRSA, antibiotic susceptibility tests, and appropriate antibiotic prescriptions.

Mathematical models have been widely used to study the spread of nosocomial bacteria in hospital settings [15-21]. In those studies patients are generally categorized into two groups: uncolonized and colonized by antibiotic-resistant bacteria. Treatments and histories of infected patients have received little attention in such models, although antibiotic exposure and treatment strategies play crucial roles among these patients. Besides, the rate of MRSA infection after colonization has been identified may be as high as 30%, and patients infected with MRSA are more likely to contaminate the hands of HCWs and the environment than colonized patients [22,23]. Moreover, the mortality rate among patients infected with MRSA may be as high as 40% [24]. Hence, in this work, we clearly separate infected patients from colonized patients to investigate factors that may influence MRSA infection. Our work focuses on MRSA infection and treatment rather than colonization and other control methods, which have been addressed in several previous modeling studies.

We first develop a baseline model, which does not incorporate the history of antibiotic usage in individuals, and study the transmission dynamics, prevalence, and persistence of MRSA infection. We then modify the model to take the history of antibiotic usage into account. In the extended model, patients are separated into two groups, with and without antibiotic history. Patients with antibiotic history are those who have previously been treated for other types of bacterial infections, or have been prescribed antibiotics outside the hospital, or have been treated for MRSA infection. In addition, we study an optimal control strategy for the use of treatment in an attempt to identify an efficient way to control MRSA infection.

## Methods

### The baseline model for MRSA transmission

**Figure 1:**
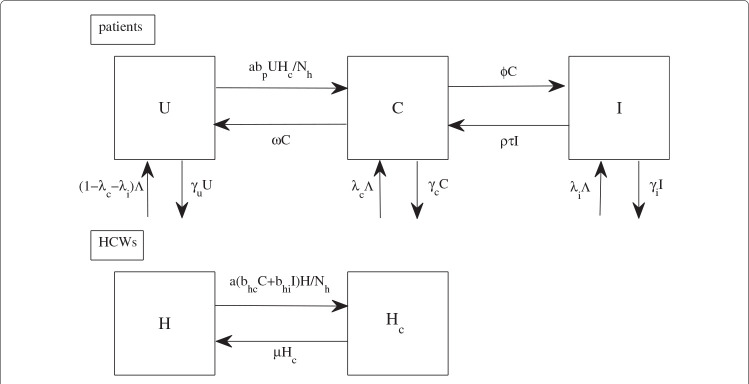
**A flow diagram for the baseline model.** The diagram shows the baseline model to describe transmission dynamics of MRSA in hospitals and the inflows and outflows of uncolonized, colonized and infectious patients (*U, C* and *I*), and uncontaminated and contaminated HCWs (*H,H*_*c*_).

To capture the transmission dynamics of MRSA among patients in hospital settings via the hands of HCWs, we employ the structure of mathematical models for vector-borne diseases [16,19]. In our case, HCWs are viewed as vectors and patients as definitive hosts. A flowchart and a table of parameter values can be found in Figure 1 and Table 1. Patients are categorized into three different groups: uncolonized (*U*), colonized (*C*), and infected (*I*) with MRSA [4]. HCWs are separated into two groups: uncontaminated (*H*) and contaminated (*H*_*c*_) with MRSA. The rates of change of these two populations are described by the following system of ordinary differential equations:

(1) $$\begin{array}{l} \dot{U}=(1-\lambda_{c}-\lambda_{i})\wedge -\frac{a}{N_{h}}b_{p}UH_{c}-\gamma_{u}U+\omega C,\\ Ċ=\lambda_{c}\wedge +\frac{a}{N_{h}}b_{p}UH_{c}+\rho \tau I-(\phi +\gamma_{c}+\omega )C,\\ İ=\lambda_{i}\wedge +\phi C-(\gamma_{i}+\rho \tau )I,\\ \dot{H}=-\frac{a}{N_{h}}b_{\text{hc}}\text{CH}-\frac{a}{N_{h}}b_{\text{hi}}\text{IH}+\mu H_{c},\\ \dot{H_{c}}=\frac{a}{N_{h}}b_{\text{hc}}\text{CH}+\frac{a}{N_{h}}b_{\text{hi}}\text{IH}-\mu H_{c}, \end{array}$$

**Table 1:** List of parameters for MRSA transmission in hospital settings

**Description**	**Symbol**	**Value**	**References**
Total number of patients	*N*_*p*_	600	
Total number of HCWs	*N*_*h*_	150	
(Patients: HCWs = 3:1)			[19]
Probability that a person is colonized by MRSA at admission	*λ*_*c*_	0.04	[24]
Probability that a person is infected with MRSA at admission	*λ*_*i*_	0.001	[25]
Average length of stay of an uncolonized patient (days)	1/*γ*_*u*_	5	[24]
Average length of stay of a colonized patient (days)	1/*κ*	7	[20]
Probability of becoming infectious	*m*_1_	0.3	[23]
Probability of decolonization	*m*_2_	0.01	estimated^∗^
Rate of progression from colonization to infection	*ϕ*	*m*_1_*κ*	
Rate of decolonization	*ω*	*m*_2_*κ*	
Discharge rate of colonized patients	*γ*_*c*_	(1 −* m*_1_ −* m*_2_)*κ*	
(including death from other causes)			
Average duration of treatment of an infected patient (days)	1/*τ*	14	[1]
Probability of a successful treatment	*ρ*	0.6	[26]
Death rate of an infected patient	*γ*_*i*_	(1−*ρ*)*τ*	
(from both disease-related and other causes)			
Total number of contacts that a patient requires per day	*a*	8	[19]
Probability of colonization after a contact with a HCW	*b*_*p*_	0.01	[27]
Probability of contamination after a contact with a colonized patient	*b*_*hc*_	0.15	[27]
Probability of contamination after a contact with an infected patient	*b*_*hi*_	0.30	estimated
Average duration of contamination (days)	1/*μ*	1/24	[27]

^*^Note that the probability of decolonization of a colonized patient is estimated by the normal time require for nasal MRSA to revert to the usual MSSA (methicillin-sensitive *Staphylococcus aureus*) which is approximately 30 days [28]. Hence, we here estimate the probability of decolonization by probability that a patient stays longer than thirty days [29].

where *N*_*h *_=* H* + *H*_*c*_. Patients are admitted to the hospital at a rate of *∧*per day; they are colonized with MRSA with a probability of *λ*_*c*_, infected with MRSA with a probability of *λ*_*i*_, or uncolonized with a probability of (1−*λ*_*c*_−*λ*_*i*_). We define *γ*_*u*_,*γ*_*c*_, and *γ*_*i *_respectively as the discharge rates of uncolonized and colonized patients, and the death rate of infected patients. Colonized patients become infected at a rate of *ϕ *per day. Decolonization of MRSA occurs at a rate of *ω* per day. Complete clearance of MRSA in infected patients does not occur without passing through the colonization state. Antibiotics have been used widely in hospitals. We assume that they are given to all infected patients at a maximum rate of *τ *to prevent further complications of infection and death due to MRSA. However, it is possible that some patients are successfully treated, while others are resistant to the antibiotics and the treatment fails. Hence, *ρ *in the model is a scaling parameter reflecting successful treatment. Because antibiotics cannot kill all the bacteria in infected patients, it is assumed that treated patients are still colonized by the bacteria after a successful antibiotic course.

Because patients colonized with MRSA are more likely than uncolonized patients to develop infection, we assume that there is no direct infection of uncolonized patients by the hands of contaminated HCWs. In mathematical models for vector-borne diseases, each mosquito bites at a constant rate (irrespective of the number of humans), whereas the rate at which humans are bitten increases in proportion the number of mosquitoes [30]. We amend these assumptions slightly: we do not assume that each HCW contacts the patients at a constant rate, but that each patient needs a constant number of contacts with HCWs per day. Under this assumption, transmission is frequency-dependent with respect to HCWs, and the rate at which HCWs contact residents increases in proportion to the number (or density) of residents. A particular HCW contacts a particular patient at a rate of *a/N*_*h*_, where *a* is the number of contacts required by each patient per day and *N*_*h*_ is the total number of HCWs. Uncolonized patients become colonized during contact with a contaminated HCW with a probability *b*_*p*_. HCWs become contaminated by contact with colonized patients with a probability *b*_*hc*_, and by contact with infected patients with a probability *b*_*hi*_.

### The extended model for MRSA transmission with antibiotic exposure

**Figure 2:**
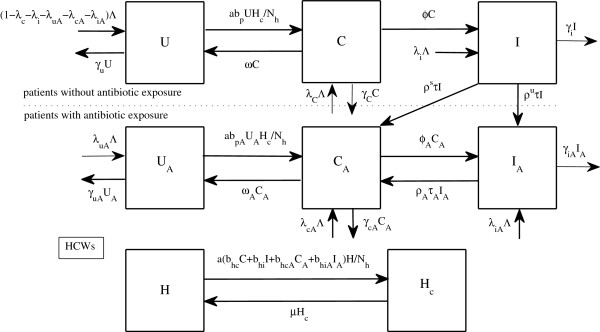
**A flow diagram for the extended model with history of antibiotic usage in patients.** The diagram shows the inflows and outflows of two groups of patients, with and without history of antibiotic usage, and HCWs.

It has been shown that patients with history of antibiotic exposure are vulnerable to skin infection and are also likely to be colonized by MRSA [12,31,32]. Furthermore, the use of antibiotics may exert a selective effect and lead to the emergence of resistant bacteria in patients and failure of treatment as a consequence. As antibiotic resistance can persist for a year, the history of antibiotic exposure of patients may become important. We therefore take this factor into account in the second model. In this extended model, treated patients either become colonized or remain infected with antibiotic history. A flowchart for this model is depicted in Figure 2. The system of equations for it is as follows:

(2) $$\begin{array}{l} \dot{U}=(1-\lambda_{c}-\lambda_{i}-\lambda_{\text{uA}}-\lambda_{\text{cA}}-\lambda_{\text{iA}})\wedge +\omega C-\frac{a}{N_{h}}b_{p}UH_{c}-\gamma_{u}U,\\ Ċ=\lambda_{c}\wedge +\frac{a}{N_{h}}b_{p}UH_{c}-(\gamma_{c}+\omega +\phi )C,\\ İ=\lambda_{i}\wedge +\phi C-(\gamma_{i}+\rho^{s}\tau +\rho^{u}\tau )I,\\ \dot{U_{A}}=\lambda_{\text{uA}}\wedge +\omega_{A}C_{A}-\frac{a}{N_{h}}b_{\text{pA}}U_{A}H_{c}-\gamma_{\text{uA}}U_{A},\\ \dot{C_{A}}=\lambda_{\text{cA}}\wedge +\frac{a}{N_{h}}b_{\text{pA}}U_{A}H_{c}+\rho^{s}\tau I+\rho_{A}\tau_{A}I_{A}-(\gamma_{\text{cA}}+\omega_{A}+\phi_{A})C_{A},\\ \dot{I_{A}}=\lambda_{\text{iA}}\wedge +\phi_{A}C_{A}+\rho^{u}\tau I-(\gamma_{\text{iA}}+\rho_{A}\tau_{A})I_{A}.\\ \dot{H}=\mu H_{c}-\frac{a}{N_{h}}(b_{\text{hc}}C+b_{\text{hiI}}I+b_{\text{hcA}}C_{A}+b_{\text{hiA}}I_{A})H,\\ \dot{H_{c}}=-\mu H_{c}+\frac{a}{N_{h}}(b_{\text{hc}}C+b_{\text{hi}}I+b_{\text{hcA}}C_{A}+b_{\text{hiA}}I_{A})H. \end{array}$$

#### Parameters for the extended model

Owing to the consequences of antibiotic exposure, patients may be at risk of having skin rashes, thrush, and gastrointestinal symptoms; and they are more likely to become infected, show a higher death rate, and have a lower probability of successful treatment because of the acquisition of resistance elements and treatment failure, if the duration of decolonization, or the length of stay are greater. Therefore, we assume that $b_{\text{pA}}\geq b_{p},b_{\text{hcA}}\geq b_{\text{hc}},b_{\text{hiA}}\geq b_{\text{hi}},\phi_{A}\geq \phi ,\gamma_{\text{iA}}\geq \gamma_{i},\rho_{A}\leq \rho^{s},\omega_{A}\leq \omega ,\gamma_{\text{uA}}\leq \gamma_{u},$ and *γ*_*cA *_≤* γ*_*c*_[8,11-14,31]. The probability of a patient having a history of antibiotic usage on admission (*λ*_*uA*_ + *λ*_*cA*_ + *λ*_*iA*_) is estimated to be 0.38 [24]. The probability of treatment failure in an infected patient (*ρ*^*u*^) (due to resistance for instance) is approximated by the probability that MSSA is replaced by MRSA in that patient [28].

## Analysis

### Steady states of the baseline model

Because the number of beds in the hospital is fixed, we assume that

$$\wedge =\gamma_{u}U+\gamma_{c}C+\gamma_{i}\mathrm{I.}$$

Hence, the total number of patients remains constant in the model. Note that the total number of HCWs is also constant.

With the admission of colonized and infected patients, there exists only a disease-present steady state $\left(U^{*},C^{*},I^{*},H^{*},H_{c}^{*}\right)$ with

$$\begin{array}{l} U^{*}=N_{p}-\frac{\lambda_{i}\gamma_{u}N_{p}}{\lambda_{i}(\gamma_{u}-\gamma_{i})+(\gamma_{i}+\rho \tau )}-\left(1+\frac{\phi -\lambda_{i}(\gamma_{u}-\gamma_{c})}{\lambda_{i}(\gamma_{u}-\gamma_{i})+(\gamma_{i}+\rho \tau )}\right)C^{*},\\ I^{*}=\frac{\lambda_{i}\gamma_{u}N_{p}}{\lambda_{i}(\gamma_{u}-\gamma_{i})+(\gamma_{i}+\rho \tau )}+\frac{\phi -\lambda_{i}(\gamma_{u}-\gamma_{c})}{\lambda_{i}(\gamma_{u}-\gamma_{i})+(\gamma_{i}+\rho \tau )}C^{*}=i_{0}+i_{1}C^{*},\\ H^{*}=N_{h}\;-\;\left\{\;\frac{ab_{\text{hi}}\lambda_{i}\gamma_{u}N_{p}+[ab_{\text{hc}}(\lambda_{i}(\gamma_{u}-\gamma_{i})+(\gamma_{i}+\rho \tau ))+ab_{\text{hi}}(\phi -\lambda_{i}(\gamma_{u}-\gamma_{c}))]C^{*}}{ab_{\text{hi}}\lambda_{i}\gamma_{u}N_{p}+\mu N_{h}(\lambda_{i}(\gamma_{u}-\gamma_{i})+(\gamma_{i}+\rho \tau ))+[ab_{\text{hc}}(\lambda_{i}(\gamma_{u}-\gamma_{i})+(\gamma_{i}+\rho \tau ))+ab_{\text{hi}}(\phi -\lambda_{i}(\gamma_{u}-\gamma_{c}))]C^{*}}\;\right\}\;\;,\\ H_{c}^{*}=\left\{\frac{ab_{\text{hi}}\lambda_{i}\gamma_{u}N_{p}+[ab_{\text{hc}}(\lambda_{i}(\gamma_{u}-\gamma_{i})+(\gamma_{i}+\rho \tau ))+ab_{\text{hi}}(\phi -\lambda_{i}(\gamma_{u}-\gamma_{c}))]C^{*}}{ab_{\text{hi}}\lambda_{i}\gamma_{u}N_{p}+\mu N_{h}(\lambda_{i}(\gamma_{u}-\gamma_{i})+(\gamma_{i}+\rho \tau ))+[ab_{\text{hc}}(\lambda_{i}(\gamma_{u}-\gamma_{i})+(\gamma_{i}+\rho \tau ))+ab_{\text{hi}}(\phi -\lambda_{i}(\gamma_{u}-\gamma_{c}))]C^{*}}\right\},\\ =\frac{h_{01}+h_{1}C^{*}}{h_{02}+h_{1}C^{*}} \end{array}$$

where C^*^ satisfies the equation

(3) $$c_{0}+c_{1}C^{*}+c_{2}C^{*2}=0,$$

in which

$$\begin{array}{l} c_{0}=\lambda_{c}[\gamma_{u}N_{p}-(\gamma_{u}-\gamma_{i})i_{0}]h_{02}+ab_{p}h_{01}(N_{p}-i_{0})+\rho \tau h_{02}i_{0},\\ c_{1}=\lambda_{c}[h_{1}(\gamma_{u}N_{p}-(\gamma_{u}-\gamma_{i})i_{0})-h_{02}((\gamma_{u}-\gamma_{c})+(\gamma_{u}-\gamma_{i})i_{1})]+ab_{p}[h_{1}(N_{p}-i_{0})\\ \;-h_{01}(1+i_{1})]+\rho \tau (h_{02}i_{1}+h_{1}i_{0})-h_{02}(\phi +\gamma_{c}+\omega ),\\ c_{2}=-\lambda_{c}h_{1}[(\gamma_{u}-\gamma_{c})+(\gamma_{u}-\gamma_{i})i_{1}]-ab_{p}h_{1}(1+i_{1})+\rho \tau h_{1}i_{1}-(\phi +\gamma_{c}+\omega )h_{1} \end{array}$$

where *N*_*p*_ is the total number of patients. Without admission of colonized and infected patients (*λ*_*c *_=* λ*_*i *_= 0), there are two steady states: disease-free (*N*_*p*_, 0, 0, *N*_*h*_, 0) and disease-present.

### The basic reproductive number (*R*_0_)

It is important to understand when an outbreak can take place, and hence to calculate the basic reproductive number (*R*_0_). In the simplest case, when only uncolonized patients are admitted (*λ*_*c *_=* λ*_*i *_= 0), we follow a method developed by [33,34] to calculate *R*_0_. Let us introduce ℱ as a vector of new infections in which ${ℱ}_{i}$ is the rate of appearance of new infections in compartment *i* (*i *= 1,2,3,4,5) and V as a vector of transfer rates by all other means in which $V_{i}=V_{i}^{-}-V_{i}^{+}$, where $V_{i}^{+}$ represents the transfer of individuals into compartment *i* and $V_{i}^{-}$ represents the transfer of individuals out of compartment *i*. To compute *R*_0_, we rearrange the order of equations and variables in (1) as *C, I, H*_*c*_*U*, and *H*.

$$ℱ=\left[\begin{array}{c} \frac{a}{N_{h}}b_{p}UH_{c}\\ 0\\ \frac{a}{N_{h}}b_{\text{hc}}\text{HC}+\frac{a}{N_{h}}b_{\text{hi}}\text{HI}\\ 0\\ 0 \end{array},\;\right]\;\text{and}\;V=\left[\begin{array}{c} (\phi +\gamma_{c}+\omega )C-\rho \tau I\\ (\gamma_{i}+\rho \tau )I-\phi C\\ \mu H_{c}\\ \frac{a}{N_{h}}b_{p}UH_{c}-(\gamma_{c}C+\gamma_{i}I)-\omega C\\ \frac{a}{N_{h}}b_{\text{hc}}\text{HC}+\frac{a}{N_{h}}b_{\text{hi}}\text{HI}-\mu H_{c} \end{array}\right].$$

Next, we define *F* and *V * as the partitioned matrices from the disease-related variables of the Jacobian matrices of ℱ and V at the disease-free steady state, respectively. Hence, we have

$$F=\left[\begin{array}{lcr} 0 & 0 & ab_{p}\frac{N_{p}}{N_{h}}\\ 0 & 0 & 0\\ ab_{\text{hc}} & ab_{\text{hi}} & 0 \end{array}\right]\;\text{and}\;V=\left[\begin{array}{lcr} \phi +\gamma_{C}+\omega & -\rho \tau & 0\\ -\phi & \gamma_{i}+\rho \tau & 0\\ 0 & 0 & \mu \end{array}\right].$$

The next generation matrix is

$$FV^{-1}=\left[\begin{array}{ccl} 0 & 0 & \frac{ab_{p}q}{\mu }\\ 0 & 0 & 0\\ \frac{ab_{\text{hc}}(\gamma_{i}+\rho \tau )+ab_{\text{hi}}\phi }{\gamma_{i}(\phi +\gamma_{c}+\omega )+\rho \tau (\gamma_{c}+\omega )} & \frac{ab_{\text{hc}}\rho \tau +ab_{\text{hi}}(\phi +\gamma_{c}+\omega )}{\gamma_{i}(\phi +\gamma_{c}+\omega )+\rho \tau (\gamma_{c}+\omega )} & 0 \end{array}\right].$$

Therefore, the basic reproductive number (*R*_0_), which is the positive and maximum eigenvalue of *FV *^-1^, is

(4) $$R_{0}=\sqrt{\frac{ab_{p}N_{p}}{\mu N_{h}}\left(\frac{ab_{\text{hc}}(\gamma_{i}+\rho \tau )+ab_{\text{hi}}\phi }{\gamma_{i}(\phi +\gamma_{c}+\omega )+\rho \tau (\gamma_{c}+\omega )}\right)}.$$

Note that some authors use the square of this term as the basic reproductive number. By Theorem 2 in [33], we conclude that the disease-free steady state is unstable if and only if *R*_0_ < 1. However, when admission of colonized and infected patients is taken into account, there is always an influx of them moving into the *C* and *I* compartments. Although *R*_0_ < 1, the spread of ARB among patients can still persists.

### Optimal control

We use optimal control techniques to study the optimal treatment rate of infected patients. The treatment rate is chosen as a control variable. We consider the following objective functional:

(5) $$J(\tau )=\text{min}\int_{0}^{T}\text{AI}(t)+E\tau^{2}(t)\text{dt}.$$

We wish to find the optimal treatment rate that minimizes the number of infected patients (A = 1) or the hospitalization costs (A=$1555 per day [35], for instance) in a limited time interval. The term *τ*^2^ is included to prevent the occurrence of a bang-bang control and also to reflect the minimization of the control itself. We follow the steps in [36] to solve the problem. The baseline model for MRSA transmission can be rewritten as follows:

(6) $$\frac{d\bar{x}}{\text{dt}}=\text{f}(\bar{x},\tau ,t),$$

where $\bar{x}=(U,C,I,H,H_{c}),\bar{x}(0)={\bar{x}}_{0}$ and $\text{f}=(f_{1}(\bar{x},\tau ,t),f_{2}(\bar{x},\tau ,t),f_{3}(\bar{x},\tau ,t),f_{4}(\bar{x},\tau ,t),$$f_{5}(\bar{x},\tau ,t))$. Introducing five adjoint variables, we have the Hamiltonian as follows

$$\tilde{H}=\text{AI}+E\tau^{2}+\overset{5}{\underset{i=1}{\sum }}\lambda_{i}f_{i}(\bar{x},\tau ,t).$$

The adjoint and transversality conditions are

$$\frac{d\lambda_{i}}{\text{dt}}=-\frac{\mathrm{\partial H}}{\partial x_{i}},\lambda_{i}(T)=0,\;\;\text{for}\;\;i=1,2,\ldots ,5.$$

Because the control is bounded, the optimality condition is

$$\begin{array}{ll} \tau =0 & \text{if}\;\;2E\tau +(\lambda_{2}-\lambda_{3})\rho I<0,\\ 0\leq \tau \leq b & \text{if}\;\;2E\tau +(\lambda_{2}-\lambda_{3})\rho I=0,\\ \tau =b & \text{if}\;\;2E\tau +(\lambda_{2}-\lambda_{3})\rho I>0, \end{array}$$

where *b* is the upper bound of the control variable. The problem is solved by the forward-backward sweep method.

We also employ the optimal control techniques to study the extended model with antibiotic exposure. We follow the similar steps with the baseline model and consider the optimal problem of the following objective functional:

(7) $$J(\tau ,\tau_{A})=\text{min}\int_{0}^{T}\text{AI}(t)+BI_{A}(t)+E\tau^{2}(t)+F\overset{2}{\underset{A}{\tau }}(t)\text{dt}$$

with the Hamiltonian

$$\tilde{H}=\text{AI}+BI_{A}+E\tau^{2}+F\tau_{A}^{2}+\overset{8}{\underset{i=1}{\sum }}\lambda_{i}f_{i}(\bar{x},\tau ,t)$$

and the following optimal conditions:

$$\begin{array}{ll} \tau =0 & \text{if}\;\;2E\tau -\lambda_{3}(\rho^{s}+\rho^{u})I+\lambda_{5}\rho^{s}I+\lambda_{6}\rho^{u}I<0,\\ 0\leq \tau \leq b & \text{if}\;\;2E\tau -\lambda_{3}(\rho^{s}+\rho^{u})I+\lambda_{5}\rho^{s}I+\lambda_{6}\rho^{u}I=0,\\ \tau =b & \text{if}\;\;2E\tau -\lambda_{3}(\rho^{s}+\rho^{u})I+\lambda_{5}\rho^{s}I+\lambda_{6}\rho^{u}I>0, \end{array}$$

and

$$\begin{array}{ll} \tau_{A}=0 & \text{if}\;\;2F\tau_{A}+(\lambda_{5}-\lambda_{6})\rho_{A}I_{A}<0,\\ 0\leq \tau_{A}\leq b & \text{if}\;\;2F\tau_{A}+(\lambda_{5}-\lambda_{6})\rho_{A}I_{A}=0,\\ \tau_{A}=b & \text{if}\;\;2F\tau_{A}+(\lambda_{5}-\lambda_{6})\rho_{A}I_{A}>0. \end{array}$$

## Results

### The baseline model for MRSA transmission

When the admission of colonized and infected individuals is not included, two situations can occur: MRSA becomes either extinct or endemic in the hospital. The threshold condition is in term of the basic reproductive number (*R*_0_) [33,34]; MRSA dies out if and only if

(8) $$R_{0}=\sqrt{\frac{ab_{p}N_{p}}{\mu N_{h}}\left(\frac{ab_{\text{hc}}(\gamma_{i}+\rho \tau )+ab_{\text{hi}}\phi }{\gamma_{i}(\phi +\gamma_{c}+\omega )+\rho \tau (\gamma_{c}+\omega )}\right)}<1.$$

**Figure 3:**
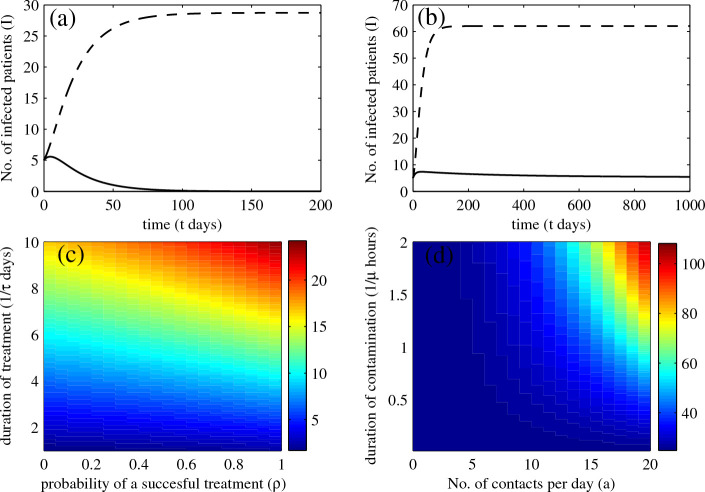
**Prevalence of MRSA infection.** Numerical solutions: (**a**) the number of infectious patients when *R*_0_ = 0.3 that MRSA is endemic when admission of colonized and infectious patients is present (dashed trace) and it dies out when there is no admission of colonized and infectious patients (solid trace): (**b**) the endemic number of infectious patients when *R*_0_ = 1.7 while admission of colonized and infectious patients is present and absent (dashed and solid traces, respectively): (**c**) the prevalence of MRSA infection in the hospital when duration of treatment and probability of a successful treatment vary: and (**d**) the prevalence of MRSA infection when duration of contamination in HCWs and the number of required contacts from patients vary.

Hence, some parameters such as *ρτa*, and *μ* may be important in controlling the persistence and spread of MRSA. In Figure 3(a), we show that when *R*_0_ < 1, MRSA dies out if there is no admission of colonized and infectious individuals, but is endemic if colonized and infected individuals are admitted. Figure 3(b) shows that MRSA is endemic when *R*_0_ > 1 irrespective of whether colonized and infected individuals are admitted.

Furthermore, we demonstrate that the prevalence of MRSA infection increases with longer treatment duration and decreases when the probability of a successful course of treatment increases (see Figure 3(c)). It also increases when the number of required contacts with patients is greater and the hands of HCWs are contaminated for longer.

### The extended model for MRSA transmission with antibiotic exposure

Because exposure to antibiotics and the history of their usage may lead to the presence of resistant pathogens, some patients may become difficult to treat by the same antibiotics and this may lead to treatment failure. The extended model, which incorporates the history of antibiotic usage, captures differences in pathogenesis and probabilities of treatment failure among patients with and without histories of antibiotic treatment.

**Figure 4:**
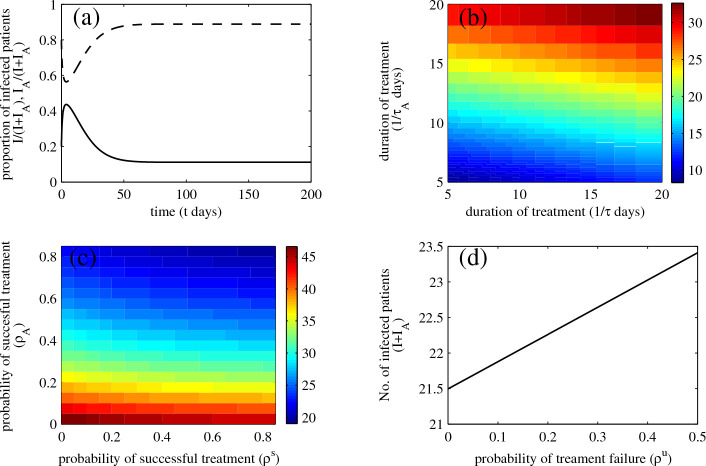
**Prevalence of MRSA infection when history of antibiotic usage is taken into account.** Numerical solutions: (**a**) the proportions of infectious patients with and without history of antibiotic exposure (dashed and solid traces, respectively): (**b**) the prevalence of MRSA infection in the hospital when duration of treatment in patients with and without antibiotics vary: (**c**) the prevalence of MRSA infection in the hospital when probability of a successful treatment in patients with and without antibiotics vary: and (**d**) the number of infectious patients according to the probability of treatment failure in the first therapy ( $\lambda_{u}=0.62,\lambda_{\text{uA}}=0.349,\lambda_{c}=0.01,\lambda_{\text{cA}}=0.03,\lambda_{i}=0,\lambda_{\text{iA}}=0.001,\gamma_{u}=\gamma_{\text{uA}}=1/5,\kappa =1/7,m_{1}=0.2,m_{1A}=0.3,m_{2}=0.01,m_{2A}=0.005,\phi =m_{1}\kappa ,\phi_{A}=m_{1A}\kappa ,\omega =m_{2}\kappa ,\omega =m_{2A}\kappa ,\gamma_{c}=(1-m_{1}-m_{2})\kappa ,\gamma_{\text{cA}}=(1-m_{1A}-m_{2A})\kappa ,\rho^{s}=0.5,\rho^{u}=0.15,\tau =1/10,\tau_{A}=1/14,\gamma_{i}=(1-\rho^{s}-\rho^{u})\tau ,\gamma_{\text{iA}}=(1-\rho_{A})\tau_{A},a=8,b_{p}=0.015,b_{\text{pA}}=0.025,b_{\text{hc}}=0.1,b_{\text{hcA}}=0.15,b_{\text{hi}}=0.25,b_{\text{hiA}}=0.3,\mu =1/(1/24)$).

Figure 4(a) shows that more patients with histories of antibiotic usage become infected over time than patients with no antibiotic history, and the infection becomes endemic in the hospital. In Figure 4(b), We vary the duration of treatment of infected patients while fixing other parameters, and investigate the effect on the prevalence of infected patients. We find that the prevalence increases with duration of treatment of infected patients, that prolonged treatment of patients with antibiotic exposure may increase the prevalence of MRSA infection more than patients without antibiotic exposure. We further investigate how the probability of a successful course of treatment influences the prevalence of MRSA infection in the hospital. Figure 4(c) shows that the prevalence is reduced when successful treatment is probable, and the success of treatment of patients with antibiotic exposure may reduce the prevalence of MRSA infection more than patients without antibiotic exposure. Hence, our findings suggest that treating infected patients with antibiotic histories as quickly and effectively as possible may help to reduce the incidence of MRSA infection in the hospital. In addition, treatment failure in infected patients without antibiotic exposure may increase the prevalence of MRSA infection (see Figure 4(d)). Hence, it may be important to establish the correct treatment at the outset infection so that infected patients will not become a reservoir for MRSA later.

### Optimal control

To seek for an efficient way to control MRSA infection and gain insight into what may affect the control measure over a limited time, we consider the treatment rate of infected patients as a time-dependent control variable and then apply optimal control techniques in order to minimize the number of infected patients (or the cost of treating them). Note that whether we minimize the number of infected patients or their hospital costs, our results suggest similar control strategies and optimal solutions.

**Figure 5:**
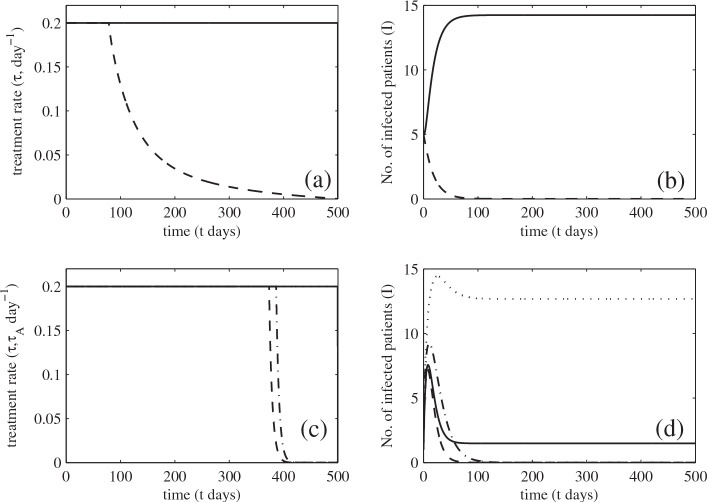
**Optimal control.** Numerical studies of optimal control strategies and solutions (*A *= 1 or 1555,*B *= 1 or 3000, *E *= 1/2, *F *= 1/2): (**a**) the optimal treatment rates for the baseline model when admission of colonized and infectious patients is present or absent (solid and dashed traces, respectively): (**b**) the number of infectious patients corresponding to the optimal treatment rates: (**c**) the optimal treatment rates for patients with and without antibiotic exposure when admission of colonized or infectious patients is present or absent (solid trace=without antibiotic exposure but with admission of colonized or infectious patients, dotted trace=with antibiotic exposure and admission of colonized or infectious patients, dashed trace=without antibiotic exposure and admission of colonized or infectious patients, dashed-dotted trace=with antibiotic exposure but without admission of colonized or infectious patients): (**d**) the number of infectious patients corresponding to the optimal treatment rates (lines are defined in (c)).

Figure 5(a) shows that to minimize the number of infected patients or their hospital costs, treatment should be given as quickly as possible at the maximum rate. This maximum effort should be prolonged when colonized and infected patients are admitted. Although the maximum rate of treatment is applied, MRSA is still endemic in the hospital when colonized and infected patients are admitted (see Figure 5(b)). However, when they are not, the treatment rate may be reduced and MRSA can be eliminated (see Figure 5(a)-(b)).

Figure 5(c) shows that the maximum treatment rate should be applied to treat infected patients in both groups (with and without antibiotic exposure) to minimize the number of such patients or their costs of treatment. However, MRSA still persists in the hospital with more infected patients with than without antibiotic history (see Figure 5(d)). When no colonized and infected patients are admitted, the maximum effort can be reduced after a certain time, but that time needs to be slightly longer for patients with antibiotic exposure (see Figure 5(c)-(d)).

## Discussion and conclusions

MRSA is a leading cause of nosocomial infections in hospitals. Hence, it is important to understand its dynamics and the factors that influence its spread in order to seek for effective ways of reducing public loss and burdens. In this work, mathematical models were used to investigate the transmission dynamics of MRSA in hospitals. We particularly focused on the prevalence of MRSA infection, the impact of treating patients with antibiotics, and how to control MRSA infection efficiently.

We first studied a baseline model for MRSA transmission that does not include the history of antibiotic usage. This general framework was developed in an attempt to understand the transmission dynamics of MRSA and how the use of antibiotics (expressed via treatment rate and probability of successful treatment) influences the prevalence of MRSA infection in the hospital. In this model, infected patients are distinguished from colonized patients, since colonization is quite widespread among humans and it does not always cause MRSA infection. By excluding the admission of colonized and infected patients, the basic reproductive number (*R*_0_) was calculated. This epidemic quantity suggests that: (1) MRSA dies out if and only if *R*_0_ < 1, and (2) MRSA colonization and infection are associated (for example) with duration of treatment, probability of successful treatment, duration of contamination in HCWs, and the number of contacts required for each patient, for example. In general, admission of colonized and infected patients can occur. When this is taken into account, our model predictions suggest that MRSA is always endemic in the hospital. Assuming *R*_0_ = 0.3, our model suggests that MRSA infection is prevalent in approximately 5% of patients.

Furthermore, we investigated how certain parameters influence the prevalence of MRSA infection. We found that the longer the duration of treatment and the lower the probability of treating infected patients successfully, the greater the resulting prevalence of MRSA infection. Hence, it is important that patients with MRSA infection are treated as quickly and efficiently as possible. Also, the longer the duration of contamination among HCWs and the greater the number of contacts required for patients, the greater the prevalence of MRSA infection. Thus, in a unit in which the number of contacts cannot be reduced (an intensive care unit, for instance), it may be important to reduce the duration of contamination among HCWs instead.

From previously published data, it has been suggested that prior exposure to antibiotics may lead to the emergence of resistant bacteria and treatment failure [12,14,32]. We extended the baseline model to incorporate the history of antibiotic usage. In the extended model, patients are separated into two groups, with and without antibiotic exposure. We found that there are likely to be more infected patients with than without antibiotic exposure in the hospital. This result may suggest that these groups of patients may be important determinants of the prevalence of MRSA infection and hospital costs because they are more difficult to treat successfully.

We investigated the relationship between the prevalence of MRSA infection and the duration and effectiveness of treatment. We found that the duration that takes to successfully treat infected patients with antibiotic exposure and the probability of treatment failure in these patients may have a greater impact on the prevalence of MRSA infection than infected patients without antibiotic exposure. Besides, as long as colonized or infected patients are admitted, our predictions suggest that MRSA persists in the hospital and consequently the maximum effort should be made consistently to treat patients as quickly and efficiently as possible. Therefore, on the basis of our findings, possible ways to reduce the prevalence of MRSA infection in the hospital include treating patients with antibiotic exposure as quickly and efficiently as possible, and screening and isolating colonized or infected patients at admission.

Because our results show that failure of treatment and more prolonged treatment may lead to a higher prevalence of MRSA infection, it may be inferred that the fewer the patients with antibiotic exposure, the less prevalent the infection. Hence, it may be important to treat patients correctly from the outset in order to reduce the number of infected patients with antibiotic exposure, and continuously to develop novel drugs to increase the efficacy of treating patients and antimicrobial susceptibility tests that may help to increase the chances of successful treatment. Note that reducing the number of colonized patients may also help to decrease the incidence of MRSA infection. However, recolonization is common, so decolonization regimens (the use of screening tests and mupirocin for instance) may be effective only in the short term during ongoing transmission. Other control measures such as hand-washing, staff cohorting, and environmental decontamination, which have not been discussed here are also very important in controlling MRSA in hospital settings.

## Competing interests

The authors declare that they have no competing interests.

## Authors’ contributions

Both authors conceived, discussed, and carried out the modeling studies and drafted the manuscript. Both authors read and approved the manuscript.
